# Hyaluronan stimulates pancreatic cancer cell motility

**DOI:** 10.18632/oncotarget.6617

**Published:** 2015-12-14

**Authors:** Xiao-Bo Cheng, Shiro Kohi, Atsuhiro Koga, Keiji Hirata, Norihiro Sato

**Affiliations:** ^1^ Department of Surgery 1, School of Medicine, University of Occupational and Environmental Health, Kitakyushu, Fukuoka 807-8555, Japan; ^2^ Department of Breast Surgery, The Fourth Affiliated Hospital of China Medical University, Shenyang, Liaoning 110032, China

**Keywords:** hyaluronan, pancreatic cancer, migration, hyaluronan synthase genes, hyaluronidase genes

## Abstract

Hyaluronan (HA) accumulates in pancreatic ductal adenocarcinoma (PDAC), but functional significance of HA in the aggressive phenotype remains unknown. We used different models to investigate the effect of HA on PDAC cell motility by wound healing and transwell migration assay. Changes in cell motility were examined in 8 PDAC cell lines in response to inhibition of HA production by treatment with 4-methylumbelliferone (4-MU) and to promotion by treatment with 12-O-tetradecanoyl-phorbol-13-acetate (TPA) or by co-culture with tumor-derived stromal fibroblasts. We also investigated changes in cell motility by adding exogenous HA. Additionally, mRNA expressions of hyaluronan synthases and hyaluronidases were examined using real time RT-PCR. Inhibition of HA by 4-MU significantly decreased the migration, whereas promotion of HA by TPA or co-culture with tumor-derived fibroblasts significantly increased the migration of PDAC cells. The changes in HA production by these treatments tended to be associated with changes in *HAS3* mRNA expression. Furthermore, addition of exogenous HA, especially low-molecular-weight HA, significantly increased the migration of PDAC cells. These findings suggest that HA stimulates PDAC cell migration and thus represents an ideal therapeutic target to prevent invasion and metastasis.

## INTRODUCTION

Pancreatic ductal adenocarcinoma (PDAC) is one of the most aggressive forms of malignant neoplasm, which is currently the 4^th^ leading cause of cancer-related deaths in western countries [1]. Several aspects contribute to this poor survival. The most prominent biological and pathological feature that characterizes pancreatic cancer progression is its early invasion to surrounding structures and metastasis to distant organs so that it often presents clinically at a late stage in the course of the disease and many patients lost the opportunity for curative surgical resection. Currently, there are no laboratory markers available to detect early stage PDAC. Painless jaundice, often the presenting symptom, tends to occur very late. Cancers of the tail of the pancreas do not involve the bile duct system, and are often even more progressed at the time of diagnosis. Additionally the complex tumor microenvironment and activated multiple aberrant signaling pathways always lead to chemoresistance of patients with PDAC [2]. Therefore, it is important to understand the molecular basis that leads to invasion, dissemination, and metastasis of PDAC in order to provide more effective therapy.

Hyaluronan (HA) is a major component of extracellular matrix (ECM). In normal physiological conditions, HA is involved in a variety of cellular processes, including cell adhesion, migration, and proliferation by interacting with specific cell surface receptors including cluster of differentiation 44 (CD44) and receptor for hyaluronic acid-mediated motility (CD168/RHAMM) [3]. HA is synthesized by HA synthases (HAS1, HAS2, and HAS3) and is degraded by hyaluronidases (mainly HYAL1, HYAL2, and HYAL3) [4, 5]. Accumulating evidence suggests that HA is involved in the aggressive phenotype of various malignant tumors [6–8]. For example, HA has been shown to promote tumor progression by enhancing cell proliferation, migration, invasion, metastasis, angiogenesis, and resistance to chemotherapeutic agents [9, 10]. Interestingly, low-molecular-weight HA (LMW-HA), rather than high-molecular-weight HA (HMW-HA), has been shown to be specifically accumulated in cancer tissues and involved in tumor progression [11–13].

Previous studies have shown increased expression of HA in pancreatic cancer [14–16]. In our previous study, we demonstrated overexpression and prognostic relevance of HA and its regulators in PDAC [17]. Recently, forced production and accumulation of extracellular HA by hyaluronan synthase (HAS) 3 overexpression have been shown to promote growth of PDAC tumor in mice [18]. These findings suggest the importance of HA in the progression of PDAC; however, only a few studies addressed its effects on the aggressive tumor phenotype, especially cell migration, in PDAC.

In the present study, we utilized different experimental models to elucidate the effect of HA on the migratory ability of PDAC cells. To modulate the endogenous HA production, we treated PDAC cells with 4-methylumbelliferone (4-MU) which is a potent inhibitor of HA synthesis [19, 20] and 12-O-tetradecanoyl-phorbol-13-acetate (TPA), a stimulator of HA synthesis through activating protein kinase C [21, 22]. We also used another model of HA induction by co-culturing PDAC cells with tumor-derived stromal fibroblasts [23]. Finally, we investigated changes in cell motility by directly adding exogenous HA to PDAC cells.

## RESULTS

### Effects of modulation of HA production on the migration of PDAC cells

**Figure 1:**
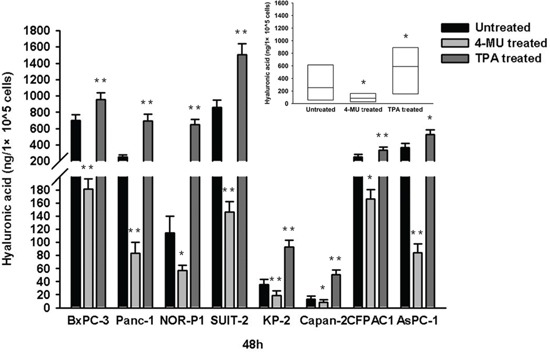
HA concentration in conditioned media of PDAC cells after treatment with 4-MU or TPA The HA concentration was significantly decreased after treatment with 4-MU but significantly increased after treatment with TPA in all the cell lines (*P < 0.05, **P < 0.01, paired t test). Each bar represents the mean ± SD of three replications.

**Figure 2:**
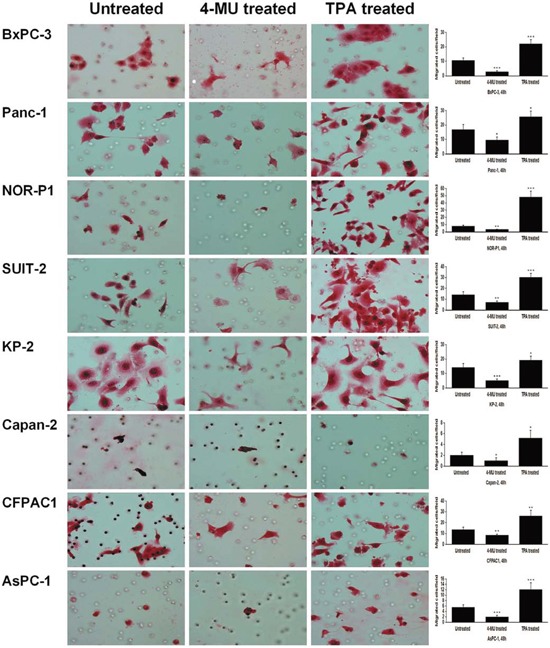
Changes in migration of PDAC cells after treatment with 4-MU or TPA by transwell migration assay (original magnification 400×) The migrating cells were significantly decreased after treatment with 4-MU and increased after treatment with TPA (*P < 0.05, **P < 0.01, ***P < 0.001, paired t test). Each bar represents the mean ± SD of six replications.

We first examined changes in migration of PDAC cells by modulating the endogenous production of HA using a known HA inhibitor, 4-MU (1000 μM), and a HA stimulator, TPA (100 ng/ml). Both drugs had no significant effects on cell viability (assessed by trypan blue dye-exclusion test) when treated at these concentrations (Supplementary Figure S1). Treatment with 4-MU resulted in a significant decrease of HA concentration, whereas treatment with TPA resulted in a significant increase of HA concentration in conditioned media of all 8 PDAC cell lines tested (Figure 1). However, treatment with 4-MU significantly inhibited the migration of PDAC cells, whereas treatment with TPA significantly enhanced the migration of PDAC cells, in both wound healing assay (Supplementary Figure S2) and transwell cell migration assay (Figure 2).

**Figure 3:**
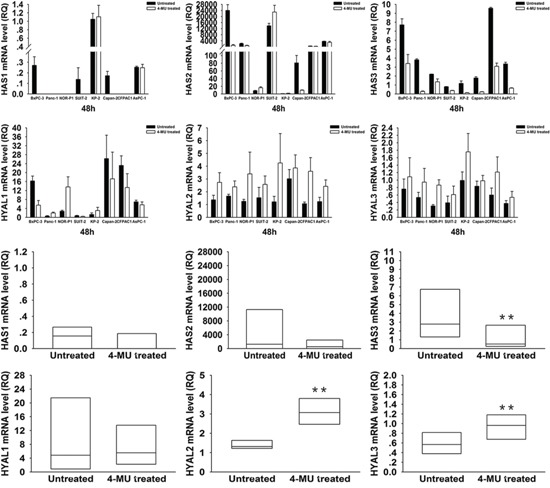
Changes in *HAS1, 2, 3* and *HYAL1, 2, 3* mRNA expressions in PDAC cells after treatment with 4-MU *HAS3* mRNA expression was significantly decreased after treatment (*P* = 0.008, wilcoxon signed ranks test), while *HYAL2* and *HYAL3* were significantly increased after treatment (*P* = 0.008, wilcoxon signed ranks test). Each bar represents the mean ± SD of three replications.

**Figure 4:**
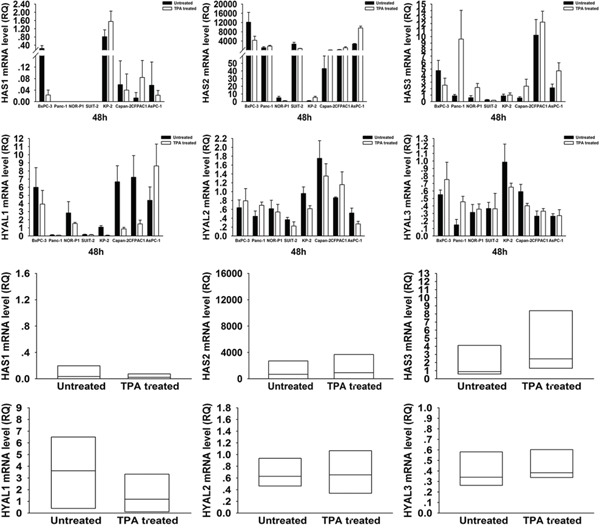
Changes in *HAS1, 2, 3* and *HYAL1, 2, 3* mRNA expressions in PDAC cells after treatment with TPA There were no significant changes in mRNA expressions of these genes after treatment (wilcoxon signed ranks test). Each bar represents the mean ± SD of three replications.

To elucidate the mechanism of HA modulation by these drugs, we investigated the mRNA expression levels of HA synthases, *HAS1∼3*, and HA degrading enzymes, *HYAL1∼3*, in PDAC cells by real-time RT- PCR (Figure 3 and 4). Treatment with 4-MU led to a significant decrease in *HAS3* but significant increases in *HYAL2* and *HYAL3* mRNA expressions. On the other hand, treatment with TPA tended to be associated with an increased mRNA expression of *HAS3* and a decreased mRNA expression of *HYAL1*.

### Effects of co-culture between PDAC cells and tumor-derived stromal fibroblasts on HA synthesis and the migration of PDAC cells

**Figure 5:**
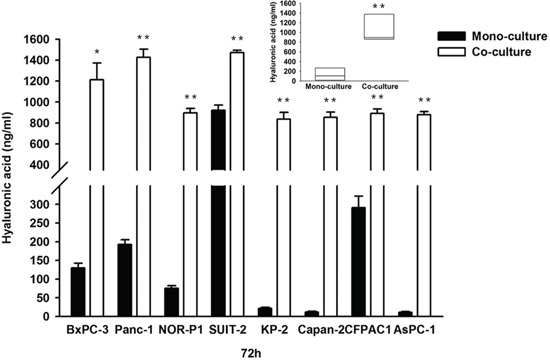
Concentrations of HA in conditioned media in mono-culture and co-culture between PDAC cells and tumor-derived fibroblasts (*P < 0.05, ** *P* < 0.01, paired t test in each cell line and median, 10.6-fold; range, 1.6- to 76.8-fold; *P* = 0.008, wilcoxon signed ranks test) Each bar represents the mean ± SD of three replications.

**Figure 6:**
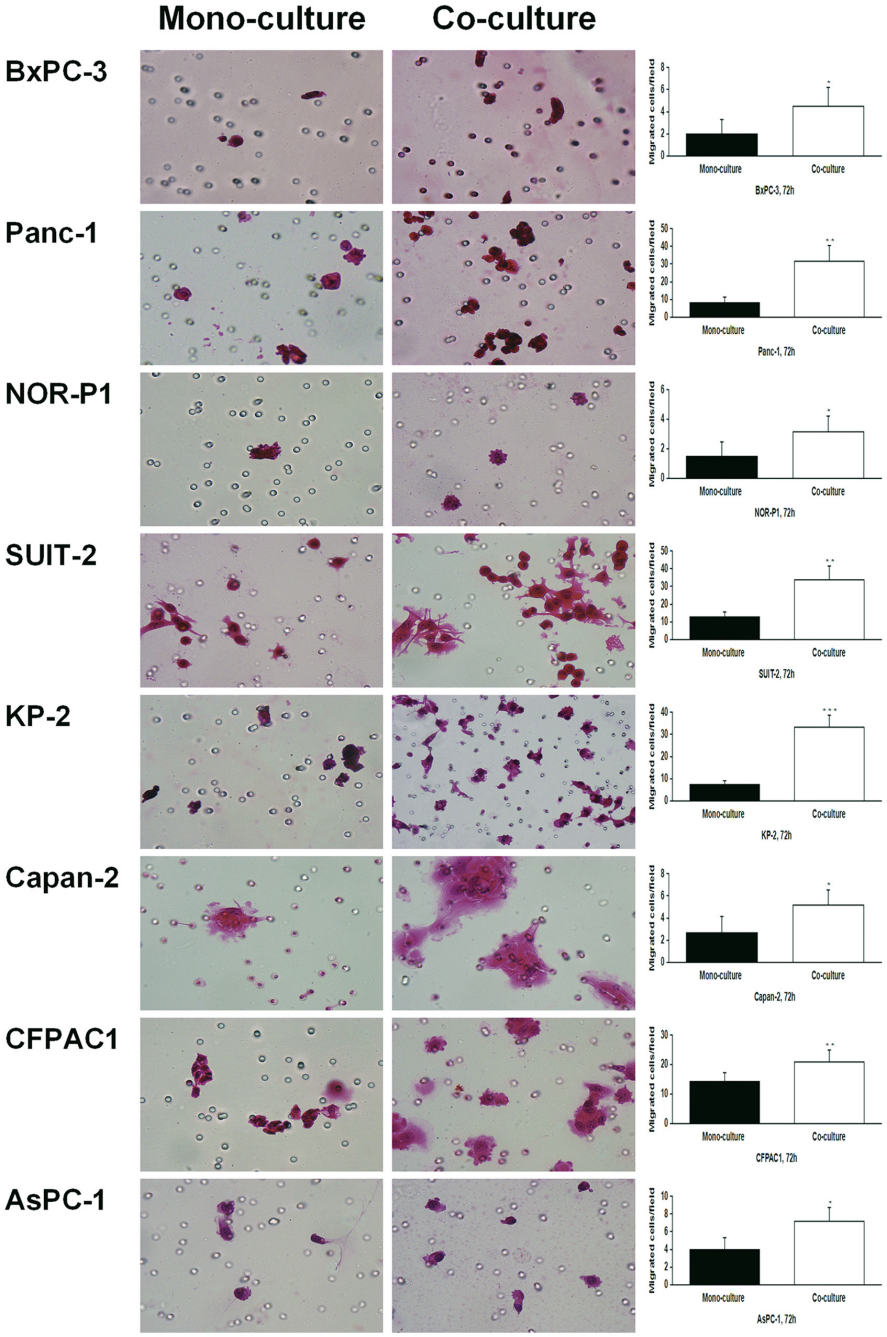
Changes in migration of PDAC cells in response to co-culture with tumor-derived fibroblasts by transwell migration assay (original magnification 400×) Co-culture significantly increased the migrating cells in all the PDAC cell lines (*P < 0.05, **P < 0.01, ***P < 0.001, paired t test). Each bar represents the mean ± SD of six replications.

We used another model, the tumor-stromal co-culture system, to determine the role of HA on the migration of PDAC cells. Co-culture of PDAC cells with tumor-derived stromal fibroblasts derived from PDAC tissue resulted in a robust increase in HA concentration in all PDAC cell lines (Figure 5). In addition, the transwell migration assay revealed that co-culture with tumor-derived fibroblasts significantly increased the migration of PDAC cells in all the tested cell lines (Figure 6).

### Effects of exogenous HA of different molecular sizes on PDAC cell migration

**Figure 7:**
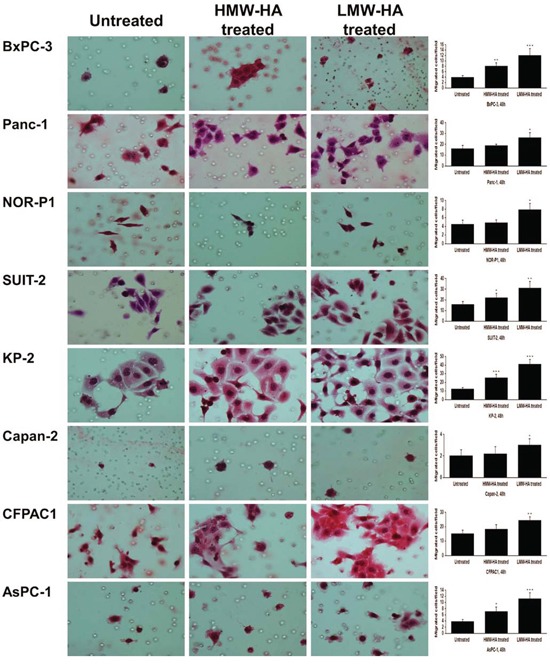
Effects of HMW-HA and LMW-HA on migration of PDAC cells assessed by transwell migration assay (original magnification 400×) The number of migrating cells was significantly increased by HMW-HA in four cell lines (BxPC-3, SUIT-2, KP-2, and AsPC-1) and by LMW-HA in all the cell lines (*P < 0.05, **P < 0.01, ***P < 0.001, paired t test). Each bar represents the mean ± SD of six replications.

To gain more direct evidence for the effects of HA on the migration, we added exogenous HMW-HA (400∼600 kDa) and LMW-HA (25∼75 kDa) to PDAC cells. In wound healing assay, the number of migrating cells was significantly increased by HMW-HA (200 μg/ml) in three cell lines (BxPC-3, KP-2, and AsPC-1) and, more robustly, by LMW-HA (200 μg/ml) in all the cell lines (Supplementary Figure S3). Similarly, transwell migration assay revealed that the migrating cells were significantly increased by HMW-HA in four cell lines (BxPC-3, SUIT-2, KP-2, and AsPC-1) and by LMW-HA in all the cell lines (Figure 7).

## DISCUSSION

In the present study, we investigated the effects of HA on PDAC cell motility. First, we used drugs (4-MU and TPA) to modulate HA production. Second, we used a co-culture system between PDAC cells and tumor-derived fibroblasts that increases HA production. Finally, we directly added different sizes of HA to PDAC cell cultures. Inhibition of HA production by 4-MU significantly decreased the migration, whereas promotion of HA production by TPA or co-culture with tumor-derived fibroblasts significantly enhanced the migration of PDAC cells. Furthermore, addition of exogenous HA, especially LMW-HA, significantly increased PDAC cell motility. These findings suggest that HA stimulates PDAC cell migration.

HA presents in a variety of molecular sizes, ranging from a few kDa to 10, 000 kDa. In the present study, we found that LMW-HA promotes PDAC cell migration more potently than HMW-HA. HA has different biological functions according to its molecular sizes. For example, excessive HMW-HA inhibits tumor growth [24, 25], whereas LMW-HA induces angiogenesis [26] and enhances motility of tumor cells [27]. Recent studies have also highlighted the importance of LMW-HA, rather than HMW-HA, in invasion and metastasis of cancers. Elevated serum levels of LMW-HA (smaller than 50kDa), but not total HA, correlated with metastasis in patients with breast cancer and decreasing the LMW-HA levels inhibited the migration and invasion of breast cancer cells [11]. Furthermore, in human colorectal cancer tissues, accumulation of LMW-HA (ranging from 6 to 25 disaccharides) in tumor interstitial fluid correlated with lymphatic invasion and lymph node metastasis [12]. These and our present findings suggest that LMW-HA, rather than HMW-HA, contributes to tumor progression by increasing cancer cell motility.

Tumor-stromal interactions can stimulate cancer progression. This is particularly true in PDAC which is characterized by a dense stroma called desmoplasia [28, 29]. In fact, the importance of tumor-stromal interactions in the aggressive behavior of pancreatic cancer is supported by several experimental evidences [30]. Among the molecules involved in the tumor-stromal interactions, HA is increasingly produced in co-culture between tumor cells and fibroblasts [23]. Interestingly, a previous study demonstrated that *HAS2* mRNA expression is significantly increased in PDAC cells in response to co-culture with tumor-derived stromal fibroblasts [31]. In the present study, co-culture between PDAC cells and tumor-derived fibroblasts synergistically increased HA production into the culture medium and enhanced PDAC cell migration. These findings suggest that increased HA production in this co-culture system might contribute, at least in part, to the increased migration of PDAC cells.

Our present results may have therapeutic implications. Because we found that treatment with 4-MU robustly attenuates the migratory ability of PDAC cells, inhibiting HA production could be an ideal therapeutic strategy to prevent invasion and/or metastasis of PDAC. In fact, inhibition of HA synthesis by treatment with 4-MU, or its derivative 4-methylesculetin, has demonstrated antitumor activity in a variety of cancers, including PDAC [32–37]. Of particular importance, 4-MU (or hymecromone) is a dietary supplement for improving liver health and can be administered orally without any toxicities [38]. A recent study showed that oral administration of 4-MU inhibits prostate cancer development, growth, and metastasis by abrogating HA signaling in treated transgenic adenocarcinoma of the prostate (TRAMP) mice [39]. Another strategy is the use of HA degrading enzyme for the treatment of HA-rich cancers. Polyethylene glycol-conjugated (pegylated) recombinant human hyaluronidase PH20 (PEGPH20) has been shown to deplete the stromal HA and substantially augment the effect of chemotherapy in PDAC animal models [40, 41]. The efficacy of PEGPH20, in combination with other drugs, is currently being tested for the treatment of advanced PDAC in clinical trials. Thus, clinical application of these HA-targeted drugs for the chemoprevention and treatment of PDAC is awaited.

In conclusion, we demonstrate that HA stimulates PDAC cell migration and thus represents a therapeutic target to prevent invasion and metastasis of this highly aggressive neoplasm.

## MATERIALS AND METHODS

### Cell culture

Eight PDAC cell lines were used: AsPC-1, BxPC-3, Panc-1, Capan-2, CFPAC1 (ATCC, Manassas, VA, USA), SUIT-2, KP-2 (JCRB Cell Bank, Osaka, Japan), and NOR-P1 (RIKEN BRC Cell Bank, Tsukuba, Ibaraki, Japan). These cell lines were recently obtained when the experiments were performed and their identities have been routinely monitored by short tandem repeat (STR) profiling. Primary fibroblasts (ike-f3) derived from PDAC tissues were kind gifts from Kyushu University. All the cell lines were maintained in Roswell Park Memorial Institute (RPMI)-1640 medium (Life Technologies, Grand Island, NY, USA) with 10% fetal bovine serum (FBS) (Life Technologies) and 1% penicillin-streptomycin (Life Technologies) at 37°C in a humidified atmosphere with 5% CO_2_.

### Reagents

4-MU, TPA, HMW-HA (molecular weight ranging from 400 to 600 kDa), and LMW-HA (molecular weight ranging from 25 to 75 kDa) were purchased from Sigma (St Louis, MO, USA).

### Trypan blue dye-exclusion assay cellular viability test

The effects of 4-MU (1000 μM) and TPA (100 ng/ml) on cell viability were analyzed using trypan blue dye-exclusion (TBDE) assays as cytotoxic measurements. After 48h, the untreated and treated cells were harvested and stained with 4% trypan blue and then counted by the LUNA™ automated cell counter (Logos Biosystems, Annandale, VA, USA) according to the instructions of the manufacturer. Cytotoxicity was determined from the number of viable cells (no color) in treated samples as a percentage of the untreated control.

### Wound healing and migration assay

About 4 × 10^5^ cells were cultured in six-well plates to form confluent monolayers and then maintained overnight in 2 ml complete medium. Then, the medium was replaced with serum-free RPMI-1640 for 12h.

For wound healing assay, the monolayer was wounded by scratching lines across the well with a standard 200 μl pipette tip. After floating cells were removed by gently washing with phosphate buffer saline (PBS), fresh medias containing 1% FBS without or with 4-MU (1000 μM), TPA (100 ng/ml), HWH-HA (200 μg/ml), or LWH-HA (200 μg/ml) were added to the wells and incubated for an additional 48h. Six representative extents of cell migration near the wound areas were photographed under light microscopy using a 5 × objective (Axio Vert 135 FL, Carl Zeiss, Germany) at the time of scratching and after 48h. The supernatant fractions of all the samples were aliquoted and stored at −80°C until assayed for the determination of HA secretion.

For migration assay, the cells were harvested and counted, and approximately 2 × 10^4^ cells were seeded in 100 μl medium with 1% FBS in the top chamber with the non-coated membrane (24-well insert; 8-μm pore size; BD Biosciences, Franklin Lakes, NJ, USA) and separately challenged without or with 4-MU (1000 μM), TPA (100 ng/ml), HWH-HA (200 μg/ml), or LWH-HA (200 μg/ml). 900 μl medium containing 10% serum was used as a chemoattractant in the lower chamber and the cells were allowed to migrate at 37°C in an atmosphere of 95% air and 5% CO2 for 48h.

All PDAC cells (2 × 10^5^/ml) in 250μl serum-free medium were separately seeded to the upper chamber and 750μl serum-free medium was added into the lower chamber as mono-culture, simultaneously, ike-f3 cells (1 × 10^5^/ml) in 750μl serum-free medium were seeded to the lower chamber as co-culture for 72h. The supernatant fractions of mono-culture and co-culture were aliquoted and stored at −80°C until assayed.

Non-migrating cells on the upper surface of the membrane were removed with a cotton swab. Migrating cells penetrated onto the lower surface of the membrane were fixed with 70% methanol, stained with hematoxylin, eosin, and air-dried. We counted the number of migrating cells in six fields randomly selected at × 400 magnification.

### ELISA-based assay for determination of HA concentration

The concentration of HA in culture medium was quantified by ELISA-based HA assay kit (R & D Systems, Minneapolis, MN, USA) according to the manufacturer's instructions.

### Real-time RT- PCR

Real-time mRNA expression analyses of *HAS1* (Hs00758053_m1), *HAS2* (Hs00193435_m1), *HAS3* (Hs00193436_m1), *HYAL1* (Hs00201046_m1), *HYAL2* (Hs01117343_g1), *HYAL3* (Hs00185910_m1), and a housekeeping gene *GAPDH* (Hs02758991_g1) for control (Applied Biosystems, Foster, CA, USA) were performed in untreated and treated cells, as described previously [42].

### Statistical analysis

Data were expressed as mean ± SD for illustration. All statistical analyses were performed with SPSS statistical software (version 21.0; SPSS, Inc., Chicago, IL, USA). Differences in HA concentration and migrating cell number between untreated and treated groups were compared using paired t test. Wilcoxon signed rank test was used to evaluate the differences in *HAS1∼3* and *HYAL1∼3* mRNA levels among untreated and treated groups. Statistical significance was accepted when *P* < 0.05. All *P* values were two-tailed; *P* values less than 0.05 were marked by one asterisk; *P* values less than 0.01 by two asterisks, and *P* values less than 0.001 by three asterisks. All experiments performed in this study were repeated three independent times.

## SUPPLEMENTARY FIGURES LEGENDS



## Abbreviations

HA: Hyaluronan
PDAC: Pancreatic ductal adenocarcinoma
4-MU: 4-Methylumbelliferone
TPA: 12-O-tetradecanoyl-phorbol-13-acetate
ECM: Extracellular matrix
CD44: Cluster of differentiation 44
CD168/RHAMM: Receptor for hyaluronic acid-mediated motility
HAS: Hyaluronan synthase
HYAL: Hyaluronidase
LMW-HA: Low-molecular-weight hyaluronan
HMW-HA: High-molecular-weight hyaluronan
TRAMP: Treated transgenic adenocarcinoma of the prostate
Pegylated: Polyethylene glycol-conjugated
PEGPH20: Polyethylene glycol-conjugated recombinant human hyaluronidase PH20
RPMI: Roswell Park Memorial Institute
FBS: Fetal bovine serum
PBS: Phosphate buffer saline
STR: Short tandem repeat
TBDE: Trypan blue dye-exclusion
ELISA: Enzyme-linked immuno sorbent assay
RT- PCR: Reverse transcription-polymerase chain reaction
CO_2_: Carbon dioxide
mRNA: Messenger ribonucleic acid
cDNA: Complementary deoxyribonucleic acid
GAPDH: Glyceraldehyde-3-phosphate dehydrogenase
SD: Standard deviation
SPSS: Statistical product and service solutions
