# Supplementary material for: Hyaluronan stimulates pancreatic cancer cell motility
Source: Oncotarget. 2015 Dec 14;7(4):4829–40. doi: 10.18632/oncotarget.6617 (PMC4826246; doi:10.18632/oncotarget.6617)
Supplement: Supplementary file 1 [file oncotarget-07-4829-s001.pdf]

SUPPLEMENTARY FIGURES LEGENDS

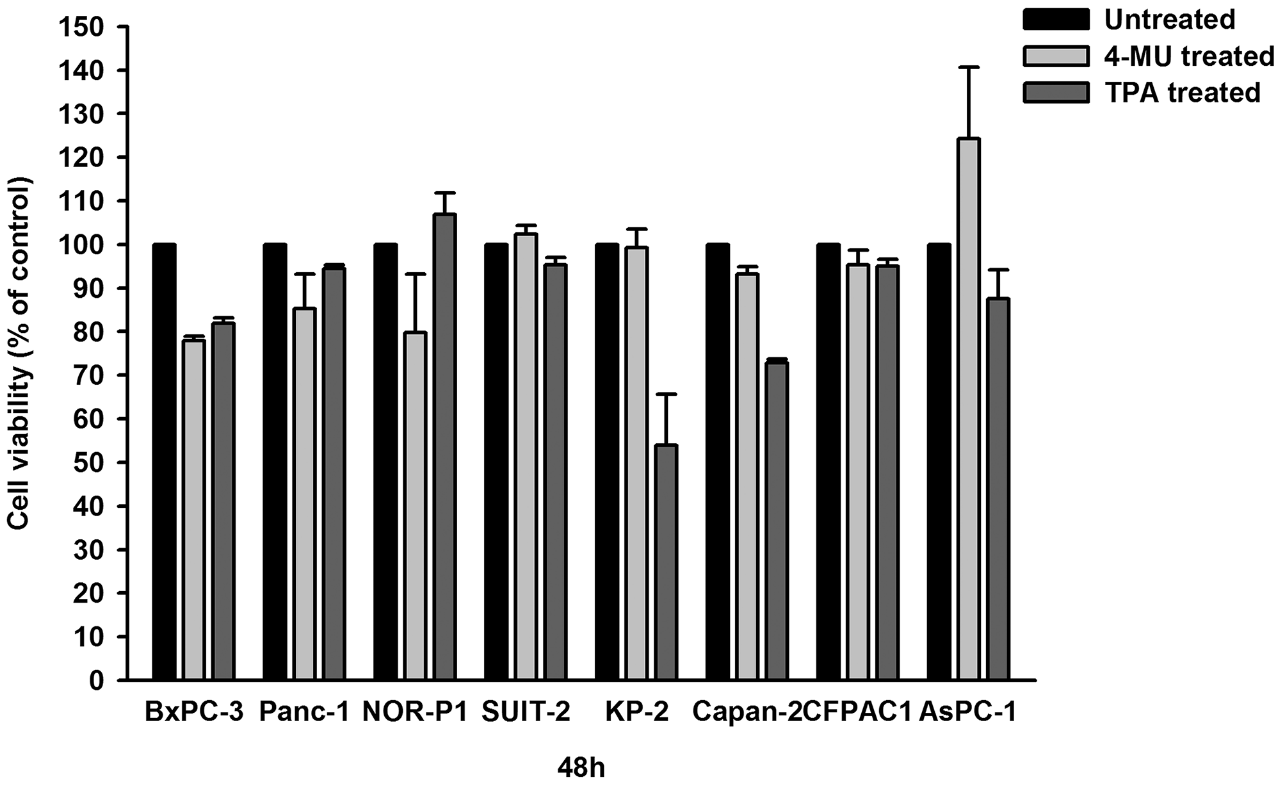

Supplementary Figure S1: Cell viability of PDAC cells after treatment with 4-MU or TPA. No significant cytotoxicity was noted after treatment with 4-MU (1000 µM) or TPA (100 ng/ml) for 48h. Each bar represents the mean ± SD of three replications.

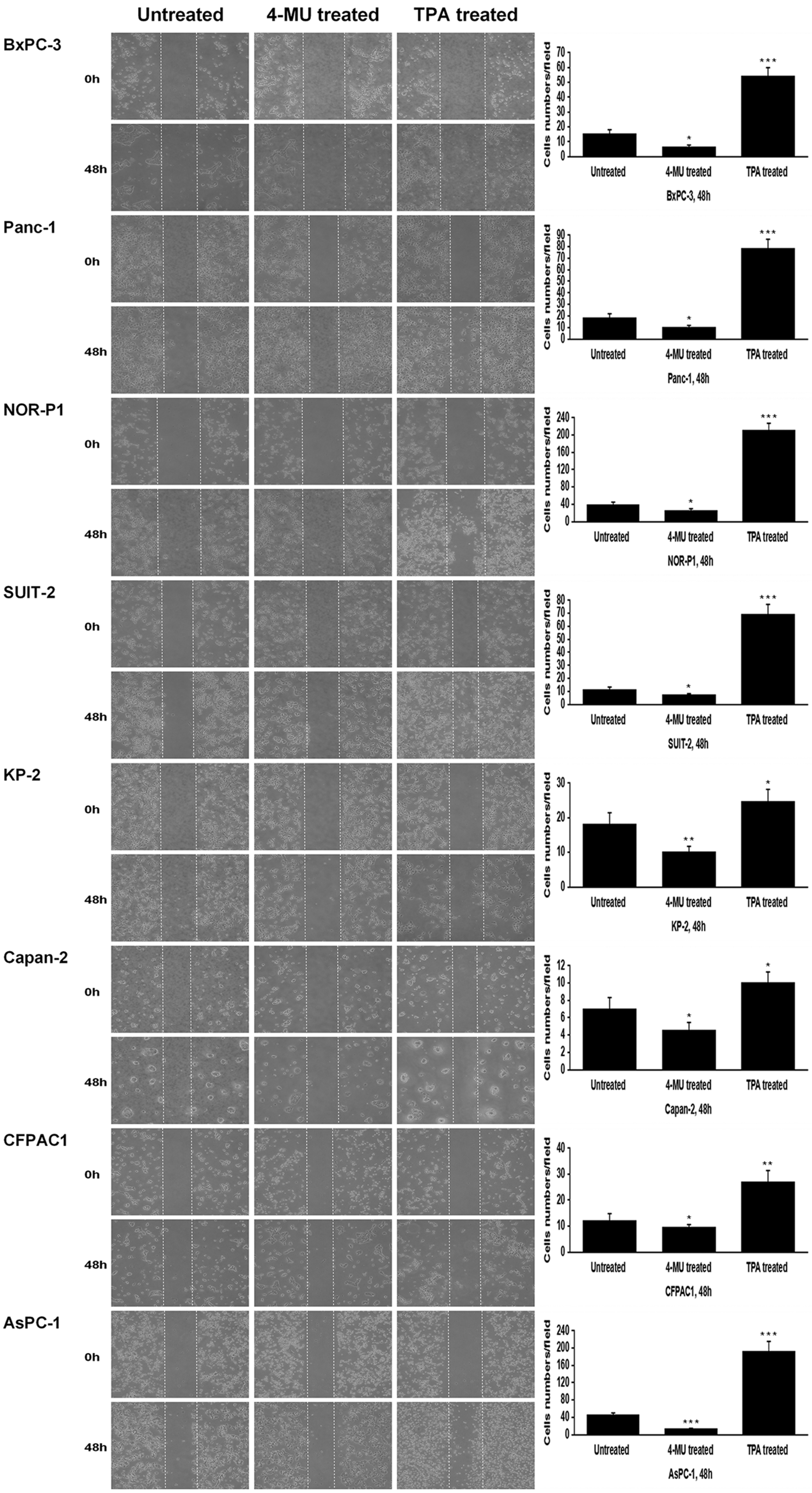

Supplementary Figure S2: Changes in migration of PDAC cells after treatment with 4-MU or TPA by wound healing assay (original magnification 5×). The migrating cells were significantly decreased after treatment with 4-MU and increased after treatment with TPA (*$P < 0.05$, **$P < 0.01$, ***$P < 0.001$, paired t test). Each bar represents the mean ± SD of six replications.

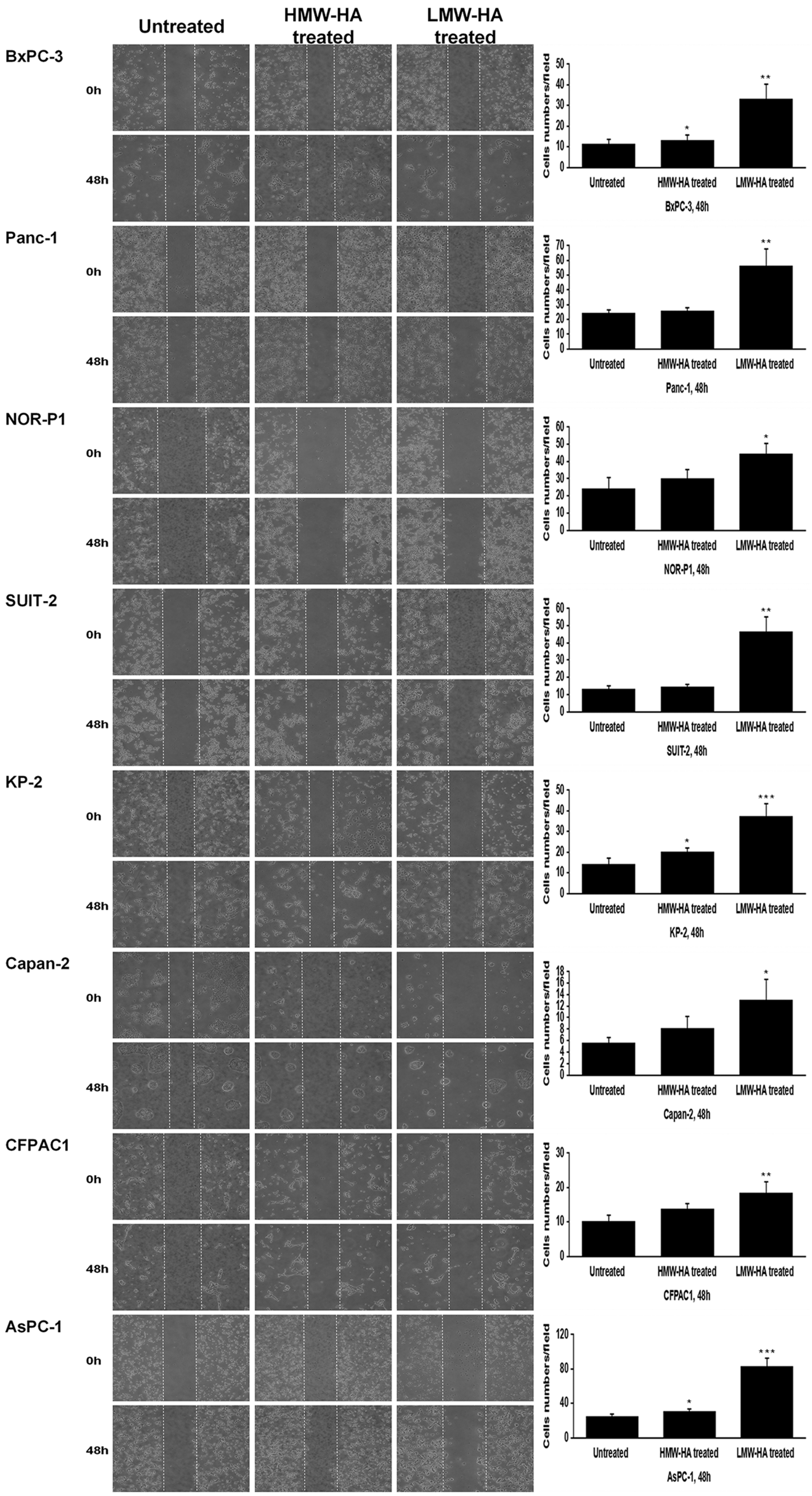

Supplementary Figure S3: Effects of HMW-HA and LMW-HA on migration of PDAC cells assessed by wound healing assay (original magnification 5×). The number of migrating cells was significantly increased by HMW-HA (200 μg/ml) in three cell lines (BxPC-3, KP-2, and AsPC-1) and, more robustly, by LMW-HA (200 μg/ml) in all the cell lines (*$P < 0.05$, **$P < 0.01$, ***$P < 0.001$, paired t test). Each bar represents the mean ± SD of six replications.
